# CBX3/HP1γ promotes tumor proliferation and predicts poor survival in hepatocellular carcinoma

**DOI:** 10.18632/aging.102132

**Published:** 2019-08-02

**Authors:** Xiaoping Zhong, Anna Kan, Wancong Zhang, Jianda Zhou, Huayong Zhang, Jiasheng Chen, Shijie Tang

**Affiliations:** 1 Department of Burns and Plastic Surgery, The Second Affiliated Hospital of Shantou University Medical College, Shantou, Guangdong 515041, P.R. China; 2 The Department of Hepatobiliary Oncology of Sun Yat-sen University Cancer Center, State Key Laboratory of Oncology in South China, Guangzhou, Guangdong 510060, P.R. China; 3 Department of Plastic Surgery, The Third Xiangya Hospital of Central South University, Changsha, Hunan 410013, P.R. China; 4 Department of Thyroid and Breast Surgery, The Fifth Affiliated Hospital of Sun Yat sen University, Zhuhai, Guangdong 519000, P.R. China

**Keywords:** CBX3, hepatocellular carcinoma, clinical significance, bioinformatics analysis, proliferation

## Abstract

HP1γ, encoded by CBX3, is associated with cancer progression and patient prognosis. However, the prognostic value and functions of CBX3/HP1γ in hepatocellular carcinoma (HCC) remain unclear. Here, we performed a bioinformatics analysis using the Oncomine, TCGA and Human Protein Atlas databases, the Kaplan-Meier plotter, and the UALCAN web-portal to explore the expression and prognostic significance of CBX3/HP1γ in patients with different cancers, including liver cancer. HCC tissues and microarrays containing 354 samples were examined using immunohistochemical staining, quantitative real-time polymerase chain reaction, and Western blotting. CBX3-overexpression HCC cell lines were tested in proliferation assays to determine the function of CBX3/HP1γ. We found that CBX3/HP1γ was upregulated in many cancers and was associated with poor prognosis. Our results also revealed that CBX3/HP1γ is elevated in HCC tissues and is associated with malignant clinicopathological characteristics. Kaplan-Meier and Cox regression analyses verified that high CBX3/HP1γ expression is an independent and significant prognostic factor for reduced overall survival in HCC patients. Moreover, *in* *vitro* functional assays showed that CBX3/HP1γ overexpression promotes HCC cell proliferation. These findings suggest that CBX3/HP1γ is an important oncogene in HCC that might act as a useful biomarker for prognosis and targeted therapy.

## INTRODUCTION

Liver cancer was the third leading cause of cancer-related mortality worldwide in 2018, and its incidence is rising, particularly in Asia and Africa [1–3]. Hepatocellular carcinoma (HCC) accounts for approximately 90% of all primary liver cancers, and prognoses remain poor for HCC patients because of the high likelihood of metastasis and recurrence [4, 5]. This low long-term survival rate is most likely attributable to the biological behavior of cancer cells [6, 7]. It is therefore important to identify novel biomarkers that are essential for HCC cell proliferation and progression and which might predict prognosis and aid in the selection of more effective targeted therapies.

Heterochromatin protein 1 gamma (HP1γ), encoded by Chromebox protein homolog 3 (CBX3), is a member of the heterochromatin protein 1 family that binds to chromatin and interacts with heterochromatin to silence gene expression [8–11]. CBX3/HP1γ can affect transcriptional activation, alternative RNA splicing, DNA damage responses, transcription elongation, and cell growth and differentiation [12–19]. Recent studies have demonstrated that overexpression of CBX3/HP1γ negatively impacts prognosis by promoting cancer development and growth in osteosarcoma [20], prostate cancer [21–23], colon cancer [24, 25], pancreatic cancer [26, 27], tongue squamous cell carcinoma [28, 29], breast cancer [30], and lung cancer [31–33]. However, CBX3/HP1γ expression and its impact on clinical pathologic parameters have not been studied in HCC patients.

In the present study, we used bioinformatics analysis to examine the expression and prognostic significance of CBX3/HP1γ in patients with different cancers, including liver cancer. We then analyzed the expression of CBX3/HP1γ in HCC tissues using quantitative real-time polymerase chain reaction (qPCR), western blotting, and immunohistochemical (IHC) staining and examined the correlations between CBX3/HP1γ, clinicopathological parameters, and prognosis in HCC patients. In addition, we performed *in* *vitro* experiments to determine the biological functions of CBX3/HP1γ. Finally, the molecular mechanisms underlying these functions were explored.

## RESULTS

### Expression and prognostic significance of CBX3/ HP1γ in various cancers

**Figure 1:**
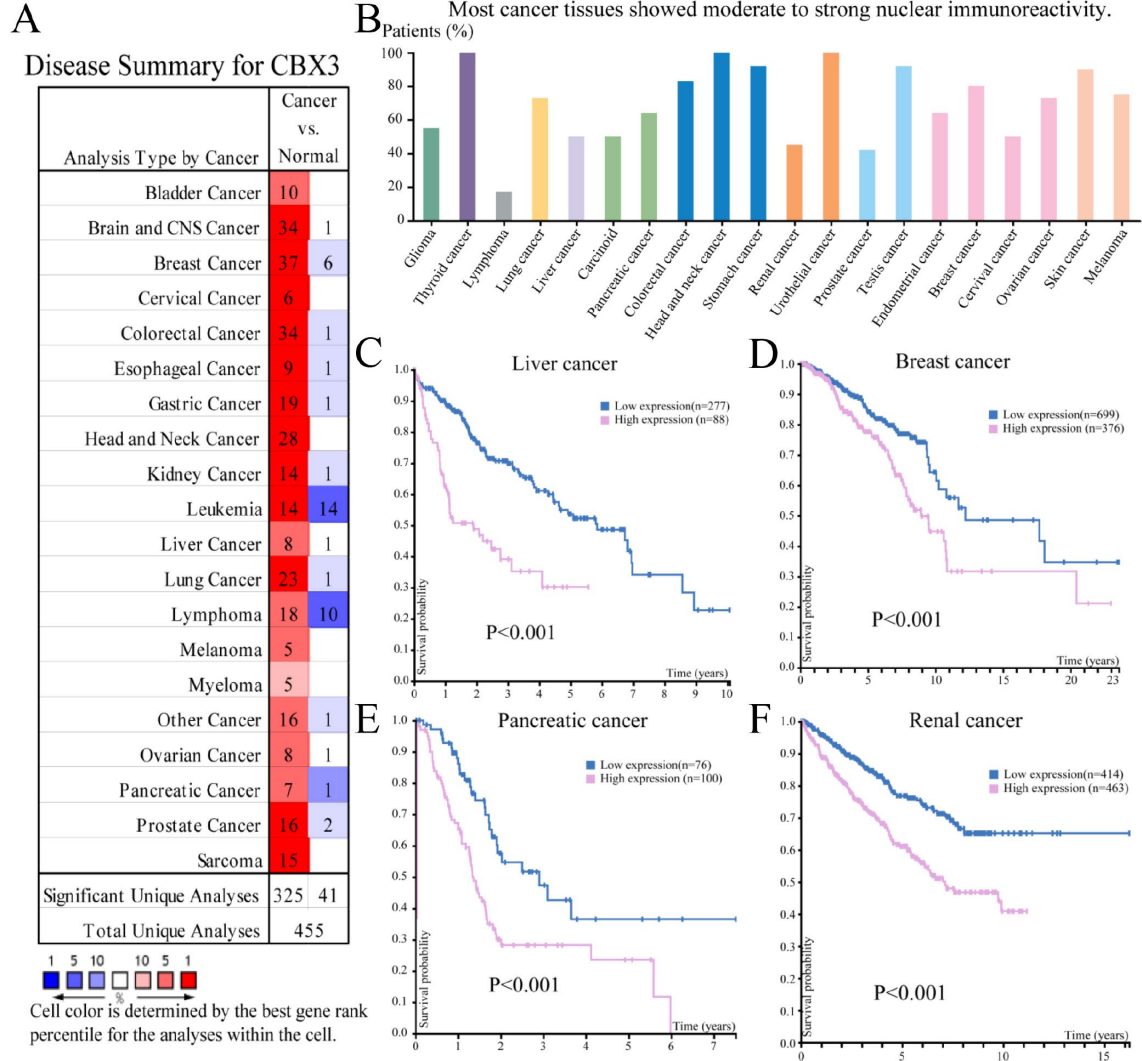
**The expression and prognostic significance of CBX3/HP1γ in various cancers.** (**A**) Transcriptional expression of CBX3/HP1γ in 20 different types of cancer (ONCOMINE database). Differences in mRNA expression were compared using Student’s t-tests. The cut-off p value and fold change values were 0.01 and 1.5, respectively, with a gene rank of 10%. (**B**) CBX3/HP1γ expression in 20 different types of cancer (the Human Protein Atlas database). High CBX3/HP1γ expression was associated with worse prognosis in liver cancer (**C**), breast cancer (**D**), pancreatic cancer (**E**), and renal cancer (**F**) (Kaplan-Meier Plot, log-rank test, P value < 0.01).

CBX3/HP1γ mRNA expression levels were examined in various cancers using the Oncomine (Figure 1A) and Human Protein Atlas (Figure 1B) databases. Most cancer tissues showed moderate to strong nuclear expression of CBX3/HP1γ. However, most lymphomas, prostate, and renal cancers displayed weak or negative staining. Bioinformatic analysis indicated that upregulated CBX3/HP1γ expression predicts poor prognosis in liver cancer (Figure 1C), breast cancer (Figure 1D), pancreatic cancer (Figure 1E), and renal cancer (Figure 1F) (Kaplan-Meier plot, log-rank test, all P values< 0.001).

### CBX3/HP1γ expression is higher in liver cancer than in normal tissues

**Figure 2:**
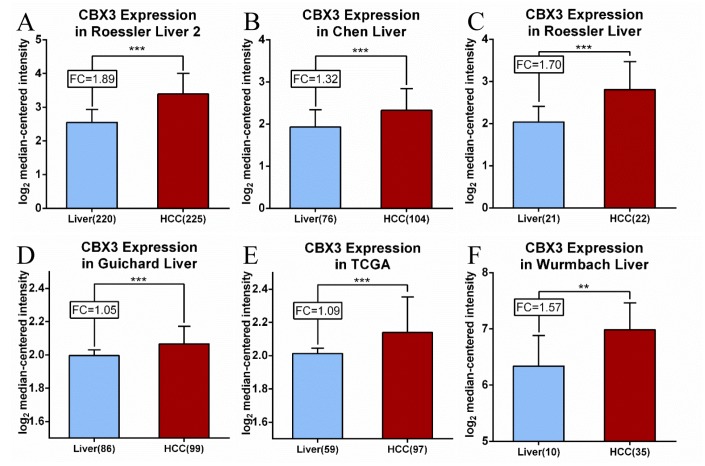
**CBX3/HP1γ mRNA expression is elevated in liver cancer compared to normal tissues.** Expression levels are shown for the (**A**) Roessler Liver 2, (**B**) Chen Liver, (**C**) Roessler Liver, (**D**) Guichard Liver, (**E**) TCGA and (**F**) Wurmbach Liver datasets. CBX3 mRNA levels are shown as log2 median-centered intensity values. **P<0.01, *** p<0.001. FC, fold change.

CBX3/HP1γ expression was compared in liver cancer and normal liver tissues in the following six datasets from the Oncomine and TCGA databases (Figure 2): Roessler Liver 2, Chen Liver, Roessler Liver, Guichard Liver, TCGA, and Wurmbach Liver. CBX3/HP1γ mRNA expression was elevated in liver cancer tissues compared to normal tissues. In the two Roessler datasets, CBX3/ HP1γ mRNA expression was 1.89-fold (p=7.91E-63) and 1.70-fold (p=2.12E-5) higher in HCC tissues [34]. Similarly, in the Chen Liver [35], Guichard Liver [36], Wurmbach Liver [36], and TCGA datasets, CBX3/HP1γ mRNA expression was increased 1.32- (p=1.85E-8), 1.05- (p=2.36E-9), 1.48- (p=6.23E-8), and 1.09-fold (p=3.93E-8), respectively, in HCC tissues.

### Upregulation of CBX3/HP1γ is associated with poor prognosis in liver cancer

**Figure 3:**
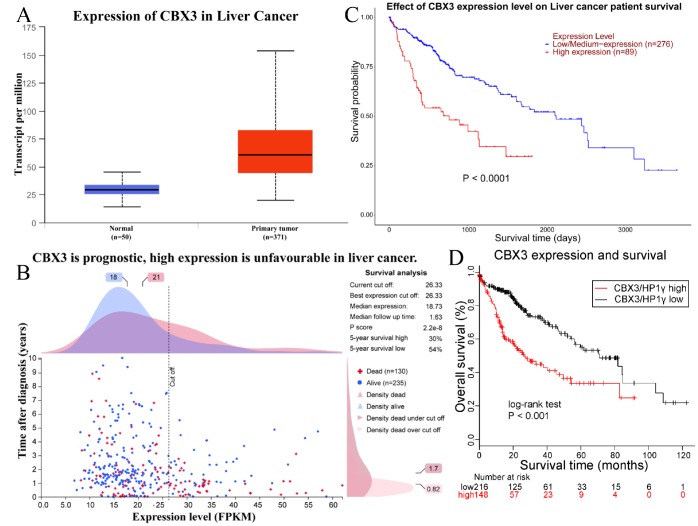
**Upregulation of CBX3/HP1γ is associated with poor prognosis in liver cancer.** (**A**) Upregulation of CBX3/HP1γ in liver cancer was confirmed in the UALCAN database (60.5 per million in primary tumor vs. 29.6 per million in normal tissue, P value = 1.62e-12). (**B**) Prognostic significance of CBX3/HP1γ expression in liver cancer in the Human Protein Atlas database. (**C**) Survival curve based on CBX3/HP1γ expression from the UALCAN web-portal (log-rank test P value < 0.0001). (D) Survival curve based on CBX3/HP1γ expression level from the Kaplan–Meier plotter survival analysis platforms (HR=2.37 (1.67-3.36), log-rank test P value = 6.9E-7).

CBX3 expression in liver cancer and normal liver tissues was verified using the UALCAN database (Figure 3). The median transcript level in primary tumors was 60.5 per million compared to 29.6 per million in normal tissue (p=1.62e-12). We explored the prognostic significance of CBX3/HP1γ expression level in liver cancer using the Human Protein Atlas database (Figures 1C and 3B), the UALCAN web-portal (Figure 3C, log-rank test p<0.0001), and the Kaplan–Meier plotter survival analysis platforms (Figure 3D, HR=2.37 (1.67-3.36), log-rank test p=6.9E-7). High CBX3/HP1γ expression was associated with unfavorable prognosis in liver cancer.

### Elevated CBX3/HP1γ expression in human HCC tissue specimens based on Western blot and qPCR analyses

**Figure 4:**
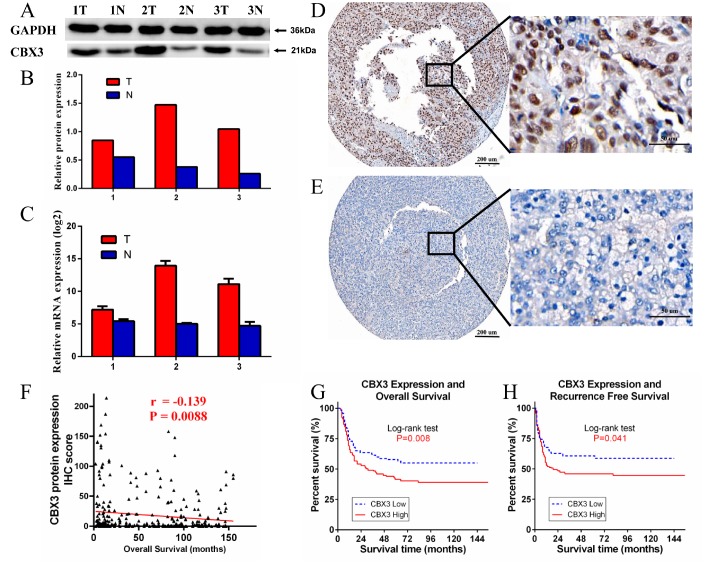
**Western blotting (WB), quantitative real-time PCR (qPCR), and immunohistochemistry (IHC) measurements of CBX3/HP1γ in hepatocellular carcinoma (HCC) tissues and HCC tissue microarrays.** (**A** and **B**) WB results show that CBX3/HP1γ expression was higher in tumor tissues than in adjacent normal tissue. (**C**) qPCR confirmed that CBX3/HP1γ expression was higher in tumor tissues. (**D** and **E**) IHC for CBX3/HP1γ protein in HCC tissue microarrays. (**D**) Representative image of strong CBX3/HP1γ staining (high expression) in tumor cell nuclei (left ×40, right ×400). (**E**) Representative image of weak CBX3/HP1γ staining (low expression) in tumor cell nuclei (left ×40, right ×400). (**F**) CBX3 expression was negatively correlated with overall survival (r = -0.139, P = 0.0088). (**G**) High CBX3/HP1γ expression was associated with worse overall survival (OS) in HCC patients (Kaplan- Meier analysis, log-rank test, P value = 0.008). (**H**) High CBX3/HP1γ expression was associated with worse recurrence free survival (RFS) in HCC patients (Kaplan- Meier analysis, log-rank test, P value = 0.041).

Next, we compared CBX3/HP1γ protein and RNA expression levels in three pairs of matched HCC tumor and adjacent non-tumor tissue using Western blots and qPCR. Both CBX3/HP1γ protein and mRNA expression were elevated in HCC tumor tissues compared to matched adjacent nonneoplastic tissues (Figure 4A–4C).

### IHC staining of CBX3/HP1γ in HCC tissue and its prognostic value in HCC patients

We performed IHC staining of HCC tissue microarrays containing samples from 354 HCC patients to evaluate the relationship between CBX3/HP1γ expression and HCC prognosis. CBX3/HP1γ protein expression was mainly located in the nucleus (Figure 4D). Based on our IHC scoring regime and the ROC curve cutoff value, patients were divided into low CBX3/HP1γ expression (negative or low expression in tumor cells; Figure 4E) and high CBX3/HP1γ expression (high expression in tumor cells; Figure 4D) groups. We then examined the relationship between CBX3/HP1γ expression levels and clinicopathological characteristics (Table 1). Among these, AFP > 20 μg/L, liver cirrhosis, tumor size > 5 cm, multiple tumors, vascular invasion, and tumor recurrence were associated with high CBX3/HP1γ expression (Chi square test P<0.05). In addition, CBX3 IHC scores were negatively correlated with overall survival (OS) times (Figure 4F).

**Table 1:** Patient characteristics.

**Variable**	**No. of patients (%)**
No. of patients	354(100)
Age:median [range], y	48.9±11.1
Gender	
Female	34(9.60)
Male	320(90.4)
HBsAg	
Negative	38(11.5)
Positive	292(88.5)
AFP: Median [range], ug/l	13759.1±33680.6
GGT: Median [range], U/l	84.9±70.3
Liver cirrhosis	
No	160(47.6)
Yes	176(52.4)
Tumor size: Median [range], cm	6.51±3.34
Tumor number	
Solitary	266(75.1)
Multiple	88(24.9)
Vascular invasion	
No	272(81.0)
Yes	64(19.4)
Tumor capsule	
No/incomplete	226(67.7)
Complete	108(32.3)
Differentiation grade^#^	
I + II	36(10.9)
III + IV	294(89.1)
Child-Pugh class	
A	334(100)
B	0(0)
TNM stage	
I	114(33.7)
II–IV	224(66.3)
BCLC stage	
0–A	288(86.2)
B–C	46(13.8)
Recurrence	
No	158(51.8)
Yes	147(48.2)
CBX3/HP1γexpression	
Low	158(44.6)
High	196(55.4)

Values are expressed as the mean ± SD or no. (%), unless otherwise indicated; Abbreviations: HBsAg, hepatitis B surface antigen; AFP, alpha-fetoprotein; GGT, gamma-glutamyltransferase. ^#^, According to the Edmondson and Steiner grading system

To confirm the correlation between CBX3/HP1γ expression levels in tumor cells and HCC prognosis, we compared OS and recurrence-free survival (RFS) between the high and low CBX3/HP1γ expression groups. RFS was defined as the time from surgery to first recurrence of HCC or death without evidence of disease. Kaplan-Meier survival analysis revealed that patients in the high expression group had poorer OS and RFS times than those in the low expression group (Figure 4G and 4H).

Univariate and multivariate survival analyses were conducted to determine whether high CBX3/HP1γ expression in tumor cells was an independent prognostic factor for HCC. Univariate survival analysis revealed that CBX3/HP1γ expression, AFP, GGT, liver cirrhosis, tumor size, tumor number, vascular invasion, and tumor capsule were significantly associated with OS. Meanwhile, GGT, liver cirrhosis, tumor size, tumor number, vascular invasion, differentiation grade, and tumor capsule were significantly associated with RFS. Multivariate survival analysis was also performed to exclude confounding factors. Hazard ratios for OS and RFS are shown in Table 2. CBX3/HP1γ expression level (HR=1.375; 95% CI 1.080–1.928; P=0.045), liver cirrhosis (HR=1.537; 95% CI 1.065–2.218; P=0.022), tumor size (HR=2.214; 95% CI 1.419–3.455; P < 0.001), tumor number (HR=3.066; 95% CI 2.116–4.441; P < 0.001), and vascular invasion (HR=2.499; 95% CI 1.720–3.633; P < 0.001) were independent prognostic factors for OS in HCC patients. In addition, liver cirrhosis (HR=1.582; 95% CI 1.112–2.250; P=0.011), tumor size (HR=2.255; 95% CI 1.461–3.482; P < 0.001), tumor number (HR=2.952; 95% CI 2.038–4.276; P < 0.001), and tumor differentiation grade (HR=3.301; 95% CI 1.535–7.102; P=0.002) were independent prognostic factors for RFS in HCC patients in this Cox regression model (Table 3).

**Table 2:** Patient baseline and correlation between CBX3/HP1γ expression and clinicopathologic characteristics in hepatocellular carcinoma (HCC).

**Clinicopathological variable**	**No. of patients**	**CBX3/HP1γ expression levels**		**P value**
		**Low expression(%)**	**High expression(%)**	
Age(years)				0.688
≤50	184	84(45.7)	100(54.3)	
>50	170	74(43.5)	96(56.5)	
Gender				0.670
Female	34	14(41.2)	20(58.8)	
Male	320	144(45.0)	176(55.0)	
HBsAg				0.718
Negative	38	16(42.1)	22(57.9)	
Positive	292	132(45.2)	160(54.8)	
AFP(ug/l)				0.021
≤20	108	39(36.1)	69(63.9)	
>20	226	112(49.6)	114(50.4)	
GGT (U/l)				0.421
≤50	130	62(47.7)	68(52.3)	
>50	206	89(43.2)	117(56.8)	
Liver cirrhosis				0.008
No	160	84(52.5)	76(47.5)	
Yes	176	67(38.1)	109(61.9)	
Tumor size(cm)				0.040
≤5	131	68(51.9)	63(48.1)	
>5	205	86(40.5)	122(59.5)	
Tumor number				0.041
Solitary	266	127(47.7)	139(52.3)	
Multiple	88	31(35.2)	57(64.8)	
Vascular invasion				0.046
No	272	131(48.2)	141(51.8)	
Yes	64	22(34.4)	42(65.6)	
Tumor capsule				0.369
No/incomplete	226	97(42.9)	129(57.1)	
Complete	108	52(48.1)	56(51.9)	
Differentiation grade^#^				0.742
I + II	36	15(41.7)	21(58.3)	
III + IV	294	131(44.6)	163(55.4)	
TNM stage				0.580
I	114	54(47.4)	60(52.6)	
II–IV	224	99(44.2)	125(55.8)	
BCLC stage				0.097
0–A	288	125(43.4)	163(56.6)	
B–C	46	26(56.5)	20(43.5)	
Recurrence				0.041
No	158	65(41.4)	93(58.9)	
Yes	147	44(29.9)	103(70.1)	

Abbreviations: HBsAg, hepatitis B surface antigen; AFP, alpha-fetoprotein; GGT, gamma-glutamyltransferase. ^#^, According to the Edmondson and Steiner grading system. Chi square test P<0.05.

**Table 3:** Univariate and multivariate analysis of the association of CBX3/HP1γ with survival and recurrence in patients with hepatocellular carcinoma.

Variables*	**OS**				**RFS**			
	**Univariate**	**Multivariable Analysis**			**Univariate**	**Multivariable Analysis**		
	**P value**	**P value**	**HR**	**95% CI**	**P value**	**P value**	**HR**	**95% CI**
Age (y) (≤ 50 vs. > 50)	0.739				0.549			
Gender (Female vs. Male)	0.553				0.254			
HBsAg (Negative vs. Positive)	0.500				0.588			
AFP (ng/ml) (< 400 vs. ≥ 400)	0.049				0.057			
GGT (U/l) (≤ 50 vs. > 50)	0.000				0.001			
Liver cirrhosis (No vs. Yes)	0.000	0.022	1.537	1.065-2.218	0.004	0.011	1.582	1.112-2.250
Tumor size (cm) (≤ 5 vs. > 5)	0.000	0.000	2.214	1.419-3.455	0.000	0.000	2.255	1.461-3.482
Tumor number (Solitary vs. Multiple)	0.000	0.000	3.066	2.116-4.441	0.000	0.000	2.952	2.038-4.276
Vascular invasion (No vs. Yes)	0.000	0.000	2.499	1.720-3.633	0.000			
Tumor capsule (No/incomplete vs. complete)	0.001				0.003			
Differentiation grade^#^ (I + II vs. III + IV)	0.673				0.002	0.002	3.301	1.535-7.102
CBX3/HP1γ expression (Low vs. High)	0.009	0.045	1.375	1.080-1.928	0.047			

*TNM stage and BCLC stage was combined with several clinical indexes such as tumor size, number and tumor thrombus; we did not enter the TNM stage and BCLC stage into multiple analysis with these indexes to avoid any bias in analysis. Abbreviations: HBsAg, hepatitis B surface antigen; OS, overall survival; RFS, recurrence free survival; AFP, alpha-fetoprotein; HR, hazard ratio; GGT, gamma-glutamyltransferase; CI, confidence interval. ^#^, According to the Edmondson and Steiner grading system.

### Stable overexpression of CBX3/HP1γ promotes growth in HCC cell lines

**Figure 5:**
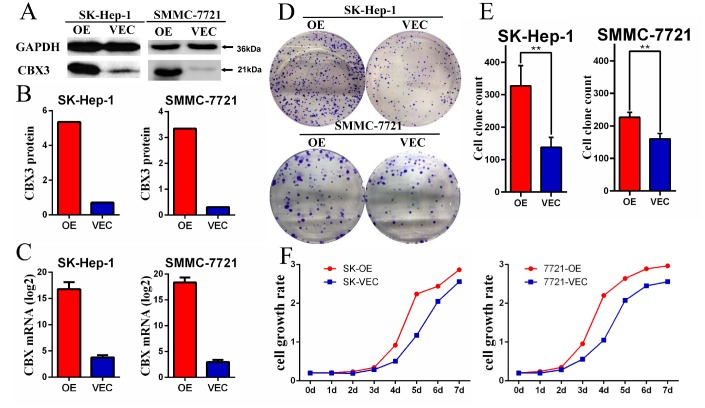
**CBX3/HP1γ was stably overexpressed in HCC cell lines and promoted cell proliferation.** (**A** and **B**) Overexpression of CBX3/HP1γ (OE) in a transfected SK-Hep-1 and SMMC-7721 cell lines compared to cells transfected with the control vector (Vec) was verified by Western blotting. (**C**) qPCR confirmed overexpression of CBX3/HP1γ mRNA in SK-Hep-1 and SMMC-7721 cell lines. (**D** and **E**) Cell colony formation assay results showing that CBX3/HP1γ overexpression promoted cell proliferation in SK-Hep-1 and SMMC-7721 cells (OE) compared to cells transfected with control vector (Vec). (**F**) CCK8 assay results showing CBX3/HP1γ overexpression accelerated cell growth rates in SK-Hep-1 and SMMC-7721 cells (OE) compared to cells transfected with control vector (Vec).

Because higher CBX3/HP1γ expression was associated with malignant clinicopathological features in HCC patients, we explored the potential biological function of CBX3/HP1γ in HCC tumorigenesis. The HCC cell lines SK-Hep-1 and SMMC-7721 were stably transfected with a CBX3/HP1γ expression plasmid (pEZ-Lv105-CBX3) or a control vector (pEZ-Lv105). Ectopic CBX3/HP1γ messenger RNA and protein expression in transfected cells was confirmed by quantitative real-time PCR and Western blot analyses, respectively (Figure 5A–5C). Functional CCK-8 and colony-forming assays were used to characterize proliferation ability in cells overexpressing CBX3/HP1γ. As shown in Figure 5D and 5E, cells overexpressing CBX3/HP1γ generated significantly more colonies than cells expressing only the vector in both the CCK-8 and colony-forming assays.

### Molecular mechanisms underlying the effects of CBX3/HP1γ

The GeneMANIA and STRING web portals were used to predict interactions between CBX3/HP1γ and other genes (Figure 6A and 6B). The results showed that CBX3/HP1γ interacts with CBX1 and CBX5, which are members of the heterochromatin protein 1 family. The CircNet database was used to predict upstream circRNAs and microRNAs of CBX3/HP1γ (Figure 6C and 6D). These results indicated that CBX3 expression may be related to circ-CBX3.7 and microRNAs such as miR-30a.

**Figure 6a:**
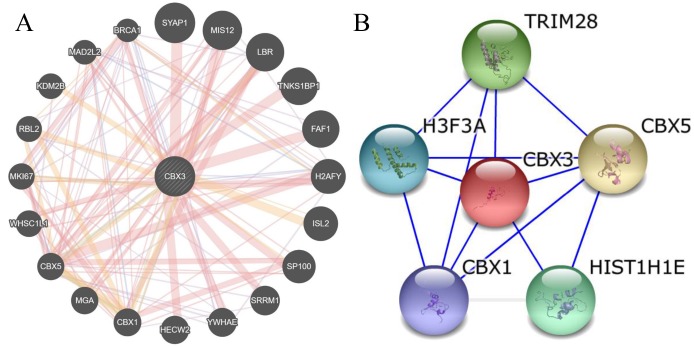
**Molecular mechanisms underlying the effects of CBX3/HP1γ.** (**A** and **B**) Interactions between CBX3/HP1γ and other genes from the web-portals GeneMANIA and STRING.

**Figure 6b:**
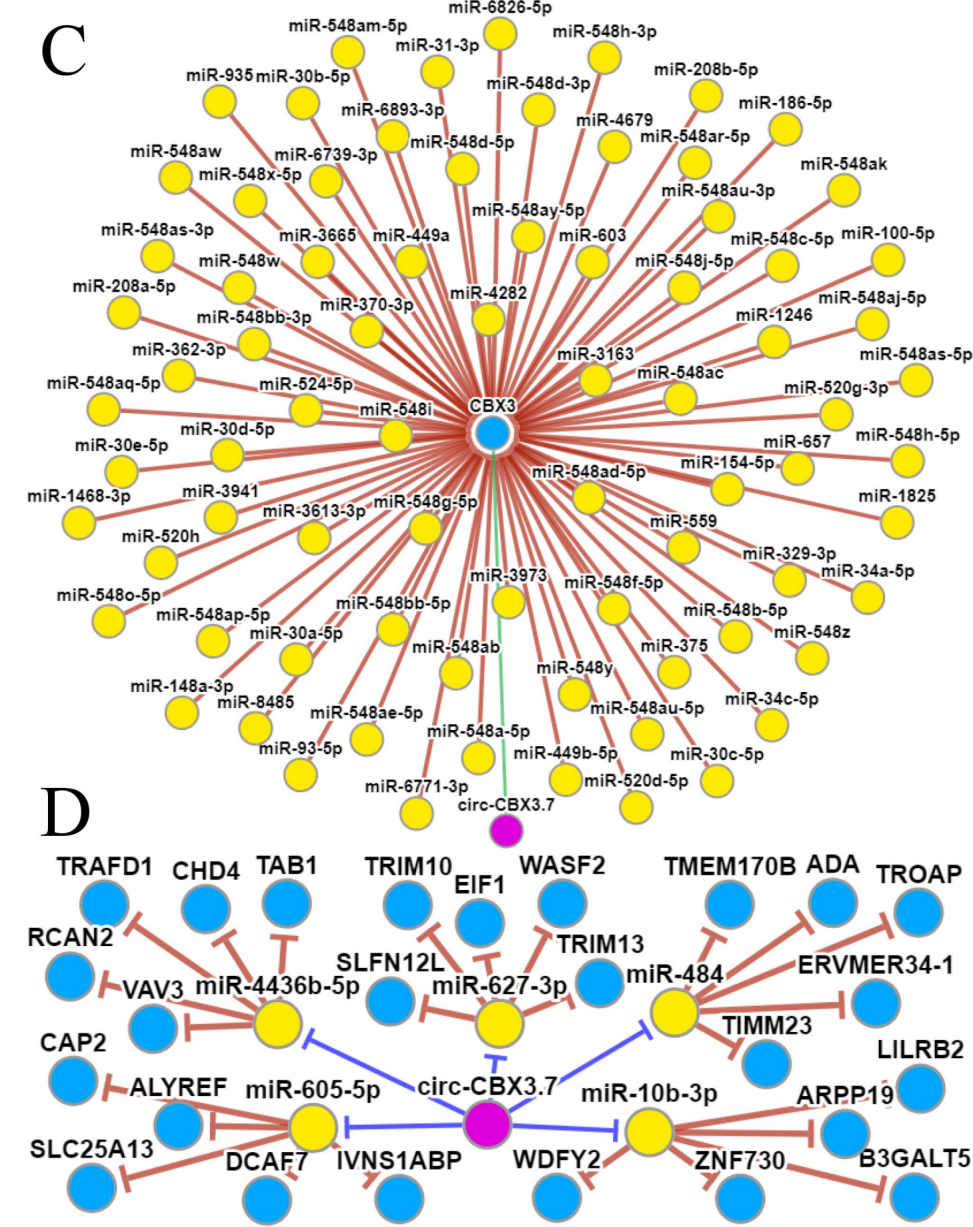
**Molecular mechanisms underlying the effects of CBX3/HP1γ.** (**C** and **D**) Upstream circRNA and microRNA of CBX3/HP1γ from the CircNet database.

## DISCUSSION

Because of tumor multiplicity and the high incidence of recurrence and metastasis after resection, long-term survival of HCC patients remains poor [2, 37]. Molecular classification and associated therapies have improved survival rates for some malignancies [38], but accurate prognosis prediction and identification of molecular treatment strategies for HCC patients has been hampered by the molecular complexity of HCC and the lack of standard tumor prediction models [3, 4, 39]. Further investigation of the molecular mechanisms involved in the development of HCC is therefore needed to identify effective prognostic and therapeutic targets.

Members of the heterochromatin protein 1 (HP1) family, also known as the chromatin binding proteins, contain an N-terminal chromodomain and a C-terminal chromoshadow domain [13, 40]. By directly binding to the promoter region of methylated H3K9 (the methyl groups of histone H3 at lysine 9), HP1 proteins regulate heterochromatin, gene expression, and developmental processes [41–43]. Abnormal expression of HP1 genes might contribute to various human diseases and cancer progression [16, 22]. CBX3/HP1γ, which is a paralog of HP1, performs various biological functions and is associated with prognosis in multiple cancers.

In the present study, we explored the expression and prognostic significance of CBX3/HP1γ in cancer patients using the Oncomine, Cancer Genome Atlas (TCGA) and Human Protein Atlas databases, the UALCAN interactive web-portal, and the Kaplan–Meier plotter survival analysis platforms. Although CBX3/HP1γ protein staining was weak in some lymphomas, prostate cancer and renal cancer, CBX3/HP1γ mRNA expression was higher in 20 types of cancer than in normal tissues (*p* value: 0.01, fold change: 1.5, gene rank: 10%; Figure 1). We also found that high CBX3/HP1γ expression was associated with poor survival in patients with liver, breast, pancreatic, or renal cancer. In order to confirm the expression and function of CBX3/HP1γ in liver cancer, multiple bioinformatics analyses were conducted. The results indicated that CBX3/HP1γ is significantly upregulated in HCC tissues compared to normal tissues in datasets from the Oncomine, TCGA, THPA, and UALCAN databases (Figure 2). This association between high CBX3/HP1γ expression and poorer prognosis in HCC patients was further confirmed using the Kaplan–Meier plotter and the UALCAN interactive web-portal (Figure 3). We then examined CBX3/HP1γ protein and mRNA expression in HCC tissues using qPCR, Western blotting, and IHC staining. CBX3/HP1γ expression as measured in those assays was associated with malignant clinicopathological characteristics, such as AFP > 20 μg/L, liver cirrhosis, tumor size > 5 cm, multiple tumors, vascular invasion, and tumor recurrence. More importantly, we demonstrated that increased CBX3/HP1γ expression in HCC tissues was associated with poorer prognoses and recurrence following curative surgery. Furthermore, univariate and multivariate analyses revealed that high CBX3/HP1γ expression in tumor cells was an independent and significant risk factor for shorter overall survival times after curative surgery.

HCC tumorigenesis is a complicated process affected by tumor multiplicity, chromosomal instability, and gene alterations. Oncogene activation and tumor suppressor inactivation are crucial steps in HCC carcinogenesis and progression. CBX3/HP1γ regulates gene expression via epigenetic silencing, and previous studies have demonstrated that up-regulation of CBX3/HP1γ occurs in a variety of cancers. Zhang *et* *al*. [28, 29] showed that CBX3/HP1γ is upregulated in tongue squamous cell carcinoma and negatively impacts prognosis by delaying the G1/S phase via p21 downregulation, which promotes tumor proliferation. Chen *et* *al*. [26, 27] found that CBX3/HP1γ promoted cell cycle transition-associated tumor progression by suppressing FBP1 in pancreatic cancer. Recent studies show that CBX3/HP1γ is upregulated in human colorectal cancer and that it promotes cell proliferation by directly regulating CDKN1A via methylation of histone H3K9 on its promoter. Moreover, miR-30a targeted CBX3/HP1γ *in* *vitro* and *in* *vivo* to specifically inhibit the growth of colorectal cancer in mouse xenograft models [24, 25]. CBX3/HP1γ is also one of the most frequently over-expressed histone reader proteins in human lung cancer, and high CBX3/HP1γ mRNA levels are associated with poorer prognosis [32, 33, 44]. Alam *et* *al*. demonstrated that CBX3/HP1γ promoted proliferation, colony formation, and migration in lung adenocarcinoma (LUAD) cells by directly repressing NCOR2 and ZBTB7A. *In* *vivo* depletion of CBX3/HP1γ suppressed K-RasG12D-driven LUAD and increased survival in mice bearing K-RasG12D-induced LUAD [31]. Itsumi [23] and Chang *et* *al*. [21] proposed that a CBX3/miR-451a/c-Myc regulatory circuitry exists in prostate cancer and that it plays a crucial role in tumor progression. Notably, Ning *et* *al*. found that overexpression of CBX family genes is significantly associated with higher clinical cancer stages and pathological tumor grades as well as reduced overall survival in HCC patients [45]. Similarly, our study showed that CBX3/HP1γ mRNA and protein expression are elevated in HCC tissues, and high CBX3/HP1γ expression was also an independent prognostic factor associated with shorter survival in HCC patients. Moreover, CBX3/HP1γ expression was associated with tumor size, tumor numbers, vascular invasion, and tumor recurrence, indicating that CBX3/HP1γ plays important role in HCC cell proliferation, invasion, and metastasis. We also demonstrated that CBX3/HP1γ overexpression promoted HCC cell proliferation. Furthermore, we explored the molecular mechanisms underlying the effects of CBX3/HP1γ using the GeneMANIA, STRING, and CircNet databases and identified potential interactions between CBX3 and other genes, micro RNAs, and circle RNAs. Overall, our results suggest that CBX3, together with other members of HP1 family (CBX1, CBX5), is regulated by miR-30a and targets downstream genes such as p21, CDK6, and CD44 to inhibit cell cycle and apoptosis, thereby promoting the growth, development, and invasion of HCC.

To the best of our knowledge, this study is the first to explore the prognostic value, cellular functions, and molecular mechanisms of CBX3/HP1γ in HCC. However, the number of patients enrolled in this study was relatively small, and the set of cellular functional assays used was limited. These results should therefore be confirmed in additional patients from different institutions. Additional studies using *in* *vitro* and *in* *vivo* assays are also needed to validate the functions and mechanisms of CBX3/HP1γ in HCC.

In summary, our bioinformatics analysis indicates that elevated Cbx3/HP1γ expression is associated with poor survival in various malignancies. Our results further suggest that high CBX3/HP1γ expression in HCC tumor cells is an independent prognostic factor associated with poorer disease outcomes in HCC patients, and that CBX3/ HP1γ plays an important role in HCC tumorigenesis and progression by promoting cell proliferation.

## MATERIALS AND METHODS

### Bioinformatics analysis

CBX3/HP1γ mRNA levels were examined in various cancers, including liver cancer, using the ONCOMINE database (http://www.oncomine.org) and The Cancer Genome Atlas database (TCGA, https://cancergenome.nih.gov/), which is a publicly accessible online database. The Human Protein Atlas database (https://proteinatlas.org/), Kaplan–Meier plotter survival analysis platforms (http://kmplot.com/), and UALCAN database (http://ualcan.path.uab.edu/) were employed to examine CBX3/HP1γ expression and survival in different cancers. Potential interactions between CBX3/HP1γ and different genes, microRNAs, and circRNAs were predicted using GeneMANIA (http://genemania.org/), STRING (http://string905.embl.de/), and CircNet (http://syslab5.nchu.edu.tw/CircNet/).

### Patients and specimens

HCC tissue microarrays containing 354 samples from patients who underwent hepatic resection for HCC at Sun Yat-sen Cancer Center between 2005 and 2013 were examined. The inclusion criteria for patient enrollment were: an absence of anticancer therapies or distant metastasis prior to the surgery; a lack of concurrent autoimmune disease, human immunodeficiency virus, or syphilis; and the availability of follow-up data. Patients with Child-Pugh scores of B or C were excluded from the study. Histologic tumor differentiation grades were assigned according to the Edmondson-Steiner grading system. The BCLC staging system and the seventh edition of the International Union Against Cancer/American Joint Committee on Cancer TNM staging system were used for staging. The clinicopathological characteristics of the patients are summarized in Table 1. Freshly resected HCC specimens and adjacent non-neoplastic liver tissues were collected from patients who had undergone hepatectomy for curative treatment of HCC at the Sun Yat-sen Cancer Center. None of these patients received any neoadjuvant therapies before surgery, including radiotherapy or chemotherapy. This study was approved by the institutional review boards of the Sun Yat-sen Cancer Center. Written informed consent was obtained from all patients, including for the use of their liver specimens for research.

### Immunohistochemistry

HCC tissue microarrays were cut into 3-μm sections for immunohistochemistry. Tissue sections were prepared for antigen retrieval using microwave treatment in citrate buffer (pH 6.0) and then incubated with a rabbit anti-CBX3 antibody (diluted 1:400 in PBS; Catalog number: 11650-2-AP, Wuhan Sanying Biotechnology, Wuhan, China) overnight at 4°C, followed by incubation with a secondary anti-rabbit HRP-conjugated antibody (Envision System, Catalog number: GK500711, Dako Cytomation, Glostrup, Denmark) at a concentration of 1:100 at 37°C for 30 min. Subsequently, the sections were washed with PBS, colorized with a diaminobenzidine (DAB) solution, and counterstained with hematoxylin. To minimize inter-observer variation, IHC staining was evaluated with a computerized image analysis platform that was constructed using the TMAJ Image application (http://tmaj.pathology.jhmi.edu) [7]. The cut-off point for low versus high expression was determined using the Youden index (sensitivity + specificity - 1) according to receiver operation characteristic (ROC) curves [38].

### Follow-up

Follow-ups consisted of physical examination, tumor marker measurement, liver biochemistry and function, blood tests, abdominal ultrasonography, and contrast-enhanced CT and were conducted for all patients less than every 1-3 months for the first year after liver operations and every 6 months thereafter for more than 60 months after treatment. The overall survival (OS) time was defined as the interval between surgery and death or between surgery and the last follow-up for surviving patients. The recurrence-free survival time was defined as the interval between surgery and recurrence or between surgery and the last follow-up for patients without recurrence. The median follow-up was 63.5 months (range 2–155 months). Of the 354 patients examined during the follow-up period, 160 patients (45.2 %) died and 157 patients (44.6 %) did not experience tumor recurrence.

### Cell lines and culture conditions

The human hepatocellular carcinoma (HCC) cell lines SK-Hep-1 and SMMC-7721 and the human embryonic kidney 293T (HEK 293T) cell line were obtained from the Liver Cancer Institute of Fudan University (Shanghai, China). All cell lines were cultured in high-glucose Dulbecco’s modified Eagle’s medium (DMEM; Gibco, Carlsbad, CA, USA) supplemented with 10% fetal bovine serum (FBS; Gibco). The cells were incubated at 37°C in a humidified incubator supplied with 5% carbon dioxide.

### Establishment of CBX3/HP1γ overexpression cells

A lentiviral CBX3/HP1γ overexpression vector was purchased from Hanbio Biotechnology Co., Ltd (Shanghai, China) and transfected into SK-Hep1 cells using Lipofectamine 2000 (Invitrogen) according to the manufacturer’s instructions. Cells transfected with empty vector were used as controls. Puromycin was used to select stable clones.

### Western blot analysis

Tissues and cells were lysed on ice in 50 mM Tris (pH 7.5), 150 mM NaCl, and 0.5% NP-40. Protein lysates were separated using 10% sodium dodecyl sulphate-polyacrylamide gel electrophoresis and then transferred to a polyvinylidene fluoride membranes. After the membranes were blocked with 5% bovine serum albumin, they were incubated with primary antibody (CBX3 or GAPDH: Catalog number: 60004-1-Ig, Wuhan Sanying Biotechnology, Wuhan, China) at 4°C overnight. The membranes were then incubated with horseradish peroxidase (HRP)-conjugated secondary antibodies at room temperature for 45 min. Protein signals were detected using enhanced chemiluminescence (Pierce, Rockford, IL, USA). Bands were quantified using ImageJ (Ver. 1.52a; NIH, Bethesda, MD), normalized to GAPDH, and expression ratios were determined.

### RNA isolation and quantitative real-time PCR

Total RNA was isolated from the tissue specimens and cell lines using TRIzol Reagent (Invitrogen Life Technologies, Shanghai, China). The isolated RNA (2 μg) was reverse-transcribed to cDNA using a SuperScript® III First-Strand Synthesis System (Invitrogen Life Technologies) according to the manufacturer’s instructions. For the qPCR assay, the cDNA was subjected to PCR amplification using SYBR Green (Toyobo, Kita-ku, Osaka, Japan) and a Roche LightCycler 480 System. GAPDH was used as an internal control. The primers used included the CBX3/HP1γ forward primer (5′-TAGATCGACGTGTA GTGAATGGG-3′), CBX3/HP1γ reverse primer (5′-TGT CTGTGGCACCAATTATTCTT-3′), GAPDH forward primer (5′- GTCTCCTCTGACTTCAACAGCG-3′), and GAPDH reverse primer (5′-ACCACCCTGTTGCTGTA GCCAA-3′).

### *In* *vitro* cell growth assays

The proliferative activity of cultured cells was determined using Cell Counting Kit-8 (CCK8) assays (Promega, WI, USA); numbers of viable cells were evaluated after 2 h of incubation in medium containing CCK8. The conversion of the tetrazolium salt WST-8 to formazan was measured at 450 nm using a plate reader. For colony formation assays, 1,000 cells were seeded in each well of a 6-well plate and cultured with DMEM (Gibco) supplemented with 10% FBS (Gibco) for 7 d. The colonies were washed twice with phosphate-buffered saline (PBS), fixed in methanol for 15 min, and stained with crystal violet for 15 min at room temperature. After the excess stain was washed out, the number of colonies was counted. Colony formation efficiency was calculated as the ratio of the number of colonies formed to the total number of cells plated.

### Statistical analyses

For continuous variables, the data are expressed as the mean ± standard error of the mean. The significance of differences between values was determined using the Student’s t-test. The chi-squared test was used to examine associations between CBX3/HP1γ expression and clinical pathological parameters. Patient survival curves were calculated using the Kaplan-Meier method and analyzed using the log-rank test. Prognostic factors were examined by univariate and multivariate analyses using the Cox proportional hazards model. All differences were deemed significant at P < 0.05. All statistical analyses were performed using SPSS software version 19.0 (SPSS, Chicago, IL).
